# Exploring problems Dutch transgender and gender diverse people experience during transition

**DOI:** 10.1080/26895269.2024.2430316

**Published:** 2024-11-21

**Authors:** Maria J. A. Verbeek, Mark A. Hommes, Jacques J. D. M. van Lankveld, Thomas D. Steensma, Arjan E. R. Bos

**Affiliations:** a Faculty of Psychology, Open University, Heerlen, The Netherlands; b Centre of Expertise on Gender Dysphoria, Amsterdam UMC, location VUmc, Amsterdam, The Netherlands; c National Institute for Public Health and the Environment, Bilthoven, The Netherlands

**Keywords:** Body dissatisfaction, concept mapping, medical treatment, misgendering, waiting list

## Abstract

***Background and Aim*:**

During transition transgender and gender diverse (TGD) people may experience various problems. This research aimed to identify the problems Dutch TGD people face during transition, to examine how frequently they encounter these problems, and to create an overview of the main problems.

***Method*:**

The concept mapping method was used to inventory problems TGD people experience during the transition process. A focus group, consisting of TGD people and care providers, formulated seventy-four such problems. Subsequently, a new group of 124 TGD participants reported how often they perceived these problems. We analyzed the frequencies of the experienced problems and the differences between groups based on gender identity and transition phase using MANOVA. The influence of age was examined with multiple regression analyses.

***Results*:**

Principal component and cluster analysis, based on these problems, revealed five clusters: problems arising from the care process, consequences of the transition in relation to yourself, consequences of the transition in relation to others, problems arising from society, and financial consequences of the transition. The most frequently experienced problems were the long waiting lists, confrontation with one’s body, and the energy the transition takes. The MANOVAs revealed that frequency scores varied with identity and phase of transition. For example, trans men and nonbinary people reported a higher frequency of experiencing problems related to body satisfaction than trans women. The frequency scores were correlated with age; younger participants scored higher on many experienced problems.

***Discussion*:**

We highlighted the problems that emerge during transition. The observed differences between groups showed that TGD people vary widely in their experiences during transition. This inventory can provide useful input for supporting TGD people during their transition and serve as a guide for the development of more TGD-inclusive policies.

## Introduction

Many terms have been used to describe people whose gender identities or gender expression are not what is typically expected for the gender assigned at birth, for example transgender, nonbinary, genderfluid, genderqueer, or bigender (Coleman et al., 2022, Riggs et al., 2015). The WPATH proposed to use the phrase *“*transgender and gender diverse*”* (TGD) to include all (Coleman et al., 2022). TGD people may choose to transition in order *“*to achieve lasting personal comfort with their gendered selves with the aim of optimizing their overall physical health, psychological well-being, and self-fulfillment*”* (Coleman et al., 2022). The transition process of TGD individuals is unique due to their widely varying personal, physical, social, and psychological situations (Coleman et al., 2022). It is a social transitioning process comprised of changes in gender expression, gender role, name, or pronoun, and it can involve a medical transition, involving gender-affirming interventions, hormone use, or surgery to achieve conformity with the desired gender identity (American Psychological Association [APA], 2015; Miller & Grollman, 2015).

Research among TGD people has mainly focused on psychological problems, experiences of rejection, mental health issues, and discrimination (Bockting et al., 2013; Moradi et al., 2016; Oorthuys et al., 2022; Tebbe & Moradi, 2016). For example, the prevalence of depression, anxiety, and loneliness exceeds the general population rates (Budge et al., 2013; Dhejne et al., 2016; Keuzenkamp, 2012) and so does the prevalence of suicide attempts among transgender people (Kohnepoushi et al., 2023; Perez-Brumer et al., 2015). TGD people experience stigmatization, harassment, and violence (Prunas et al., 2018; Stotzer, 2009; Verbeek et al., 2020) in familial situations (Lev, 2004; Von Doussa et al., 2020), at school (Pascale & DeVita, 2024), in sports (Hargie et al., 2017; Kamasz, 2018), in public (Watson et al., 2019), at work (Budge et al., 2010; Schilt, 2006; Serano, 2007; Yavorsky, 2016), in a religious environment (Moon et al., 2019), and in healthcare (Cicero et al., 2019; Johnson, 2015). They also experience misgendering, which refers to language that does not reflect the gender with which a person identifies (Coleman et al., 2022).

Additional research has focused on specific experiences, such as the emotional experiences of loss of social support and fear of exclusion from family, friends, and partners during transition (Budge et al., 2013; De Bekker et al., 2019; Fuller & Riggs, 2018; Lewis et al., 2023; Puckett et al., 2020). De Bekker et al. (2019) discussed the uncertainty about the start of counseling in specialized gender care centers, citing the experiences of Dutch TGD people with psychological problems and suicidality during transition. Carlström et al. (2021) detailed the challenges TGD individuals encounter when interacting with healthcare professionals, for example, the lack of respectful treatment. Cicero et al. (2019) concluded that transgender adults experience obstacles when accessing health care, such as lack of awareness by health care professionals and problems with health insurance. Few studies have explored the unique experiences of TGD people during various stages of transition. For instance, individuals may have to decide on surgery based on their financial situation (Riggs et al., 2015), experience an increase in libido as a result of hormone treatment (Weber-Main, 2011), or experience misgendering after their coming out (Chang et al., 2023; Mitchell et al., 2021).

The mentioned experiences, however, do not fully reflect the situations that are difficult to deal with during transition. With the present research we tried to fill this gap and we systematically explored the problems that Dutch TGD individuals experience during various stages of transition. A problem *“*is generally considered to be a task, a situation, or person which is difficult to deal with or control due to complexity and intransparency*”* (Seel, 2012). This exploratory research focused on practical and useful purposes. The aim of the current study was to identify the problems, to examine the frequency in which these problems were encountered and to inventory the most important problems. This inventory can provide useful input for supporting TGD people during their transition and serve as a guide for the development of more TGD-inclusive policies.

We examined possible differences between trans men, trans women, and nonbinary people related to these problems. Tatum et al. (2020) found that trans men, trans women, and nonbinary people go through distinct developmental trajectories. They are affected differently by cisgenderism, a form of stigma that denies, ignores, and marginalizes genders other than the assigned gender at birth (Riggs et al., 2015). We also expected younger TGD people to react differently from older TGD people during the transition. Puckett et al. (2022) found that younger TGD people showed higher levels of anxiety, internalized stigma, and depression, compared to older TGD people. We examined possible differences between the phases of transition as well. The experienced situations may vary; for example, in the pretransition phase, no surgery will take place. In study 1 we explored the problems using focus group methodology based on concept mapping. In study 2, we examined how frequently TGD people experienced these problems using online questionnaires.

## Study 1

### Method

Concept mapping is a mixed-method approach, combining qualitative components with quantitative analysis (Kane & Trochim, 2006). The concept mapping process includes statement generation through brainstorming, prioritizing, and clustering to create a concept map, a diagram that delineates relationships between concepts. We used the software package Ariadne 3.0 to automatize and visualize the concept mapping process (Severens, 2015). The involvement of TGD people as stakeholders in this process was considered to be of great importance because of their expertise concerning the transition. (Torrance, 2012).

### Researchers

Five researchers contributed to the concept mapping process and analysis for this study. The first author is a female PhD student in clinical psychology. Her main research focus is on (self)stigmatization during transition. The second author is an assistant professor in clinical psychology, a psychologist, and as a trans man, he is an advocate for TGD people. The third author is a male psychologist, psychotherapist, sexologist, and professor of clinical psychology. The fourth author is a male child and adolescent health psychologist working at a Dutch expertise center for gender dysphoria. The fifth author is a male professor in clinical psychology, specialized in stigmatization research.

### Participants

From April 2018 to September 2018, TGD participants were recruited through flyers and posters distributed at Center of Expertise on Gender Dysphoria of the Amsterdam UMC and two psychology practices specialized in gender dysphoria. Eligibility for participation in the study was evaluated at the end of the application period. We selected participants based on their gender identity, phase of transition, age, and/or expertise to create a group with a balanced mix of relevant expertise. Eligible participants were invited to attend a focus group meeting in September 2018. The focus group (*N*** **=** **13) consisted of TGD people (*n*** **=** **9), health care providers (*n*** **=** **3) and an expert (posttransition trans man and care provider). A more proportionate distribution between trans men and trans women was aimed for, but unexpected cancellations and a limited number of substitutes made the final group less heterogeneous (see Table 1).

**Table 1.** Participants characteristics.

		*n*	*M (SD)*
Participants	Trans men	2	
	Trans women	5	
	Nonbinary	2	
	Expert (trans man)	1	
	Care providers (women)	3	
Phase of transition	Pretransition	3	
	During transition	3	
	Posttransition	3	
Age	Trans women		59.6 (1.7)
	Trans men		42 (21.0)
	Nonbinary		33 (7.0)

### Concept mapping procedures

**Figure 1.**
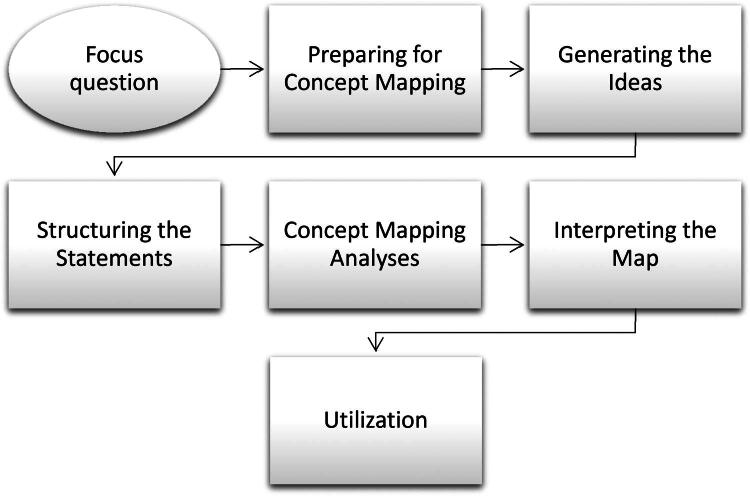
An overview of the concept mapping process adapted from “concept mapping for planning and evaluation” by Kane and Trochim (2006).

The concept mapping procedures included the following steps (see Figure 1).

#### Focus question

The focus question: “What difficult situations are TGD people confronted with during the transition process?” was formulated by the research team prior to the brainstorm session.

#### Preparing for concept mapping

Prior to the brainstorm session, participants received a program overview *via* e-mail with the focus question, a statement of informed consent, and information about travel reimbursement. Separately, they received a link *via* e-mail to log in on the website of the software program, Ariadne 3.0, for visualizing the results of the brainstorm sessions *via* concept mapping (Severens, 2015). Signed informed consent was obtained from all participants during the brainstorm session.

#### Generating the ideas

Following an introduction to the concept mapping theory, during the brainstormsession, the research team encouraged the participants to freely associate with the focus question and formulate a statement. Each participant, in turn, provided a statement, and the research team ensured the formulation of the statements was clear and specific. A research team member entered the statements (*N* = 74) directly in Ariadne 3.0. After an hour, content saturation was achieved (i.e. new statements did not yield novel content) and the brainstorm session was terminated. Thereafter, the statements were discussed briefly and, if necessary, clarified or rephrased.

#### Structuring the statements

After the brainstorm session, participants received instructions to prioritize the statements individually. Participants rated the priority of each statement on a scale from 1 (*not important*) to 5 (*very important*) using the software program Ariadne 3.0. All statements had to be equally distributed over the five rating options. Next, the participants were provided with the original list of statements to place them into clusters with related content using Ariadne 3.0. No more than 10 clusters could be made; a cluster could exist of only one statement.

#### Concept mapping analysis

**Figure 2.**
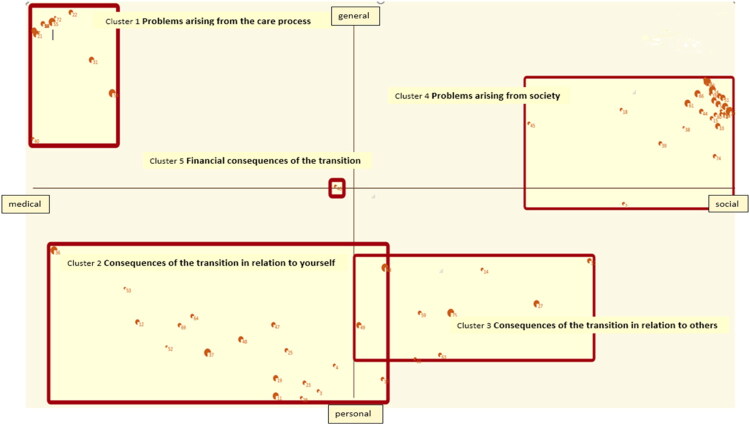
Concept map and clusters.

Ariadne 3.0 was used to analyze the data using principal component analysis and cluster analysis to identify each statement as a distinct point on a map. According to the prioritization, Ariadne 3.0 was used to calculate the relative importance per cluster and per statement. A concept map was made: a two-dimensional graph in which the clusters represented the concept (see Figure 2). The frequency with which the statements were placed in the same cluster determined their proximity within the concept map.

#### Interpreting the map

Ariadne 3.0 was used to generate a statement list that included the means and standard deviation of the priority of each statement and cluster (see Appendix A, Statements per Cluster). This procedure revealed five clusters, containing statements that belong together. Rotation was applied to get a clearer picture of the position of the clusters relative to the axes (see Figure 2). The axes were labeled by the research team: the horizontal axis appeared to represent the *medical* aspects of the transition on the left and the *social* aspects on the right. The upper half of the vertical axis appeared to represent the *general* aspects of the transition, whereas the lower half represented the *personal* aspects.

The research team interpreted and named the clusters (see Table 2). Cluster 1 *Problems arising from the care process* is a cluster about practical matters and consequences concerning the medical process of the transition. The statements pertained to the healthcare system, encompassing issues like treatment waiting times, the dependence of care providers, and the absence of psychological aftercare. Cluster 2 *Consequences of the transition in relation to yourself* focused on the impact of the transition on the individual and the consequences of medical treatment, such as diminished sexual desire, decreased fertility, and confronting one**’**s own body. Cluster *3 Consequences of the transition in relation to others* encompassed statements about relationships, including concerns of partners**’** reaction and strategies for communicating to a date that one is undergoing a transition. Other statements were about finding suitable clothing and wondering what to wear. One statement was about the fear of participating in swimming activities. There appeared to be some overlap between clusters 2 and 3 considering the loss of friends (see Appendix A, statements 3 and 27). Cluster 4 *Problems arising from society* was about problems TGD people may experience in society. Some statements were formulated from the point of view of the TGD individual such as “choosing a toilet is difficult in the beginning of transition” or “being discriminated against” (see Appendix A, statements 2 and 73). Other statements reflected societal expectations that TGD people encounter, such as the expectation of stereotypical gender norms or the expectation that one will explain one**’**s sexual orientation (see Appendix A, statements 34 and 51). The statements also included the prevalent misconceptions about TGD identity; individuals hold specific beliefs about the potential harm caused by TGD people. Cluster 5 was concerned with financial issues related to the transition.

**Table 2.** Number of statements and mean ratings per cluster.

Cluster	*n*	*M*
1. Problems arising from the care process (CARE)	15	3.52
2. Consequences of the transition in relation to yourself (SELF)	17	3.28
3. Consequences of the transition in relation to others (OTHER)	11	2.99
4. Problems arising from society (SOC)	30	2.67
5. Financial consequences of the transition (FIN)	1	3.46
Total	74	

The mean ratings per cluster show the importance of the clusters scored by members of the focus group (Table 2).

## Study 2

Following the concept mapping process, we examined the extent to which a broader group of TGD individuals experienced these problems. Therefore, we composed a list of the most important problems, termed the Transgender and Gender Diverse Problems (TGDP) list. This TGDP list was used (a) to measure the frequency with which the TGD participants experienced these problems and (b) to investigate possible differences between subgroups related to gender identity, transition phase, and age.

## Introduction

In this exploratory study, we aimed to answer the following research questions: 1 How often are problems experienced by TGD participants; 2 Are there differences between the experiences of trans men, trans women, and nonbinary people with regard to the experienced frequency of the problems; 3 Are there differences between the TGD participants with regard to the experienced frequency of the problems in distinct phases of the transition; and 4 Is age associated with the experienced frequency scores related to the problems?

### Reduction of statements

The research team composed the TGDP list by reducing the number of statements (see Appendix A) based on the mean scores from step 3 in Study 1. We selected the statements based on their highest scores, using a cutoff score of *M*** **=** **3.00. Some statements were (partially) similar, e.g. statement 10 “The medical transition process takes a long time” and statement 35 “Slow progress in treatment.” We deleted statement 35. Some statements were selected because they were considered exemplary for problems TGD people may encounter despite their lower mean score, for example the statements 1 “At the start of transition buying clothes is difficult” (*M*** **=** **2.38) and 29 “It is difficult to change dressing rooms at the gym” (*M*** **=** **2.38). The only statement in cluster 5 “Financial consequences of the transition” was added to cluster 4 for the financial consequences are mostly related to social situations, for example losing a job. In the end, we selected 47 statements for the Transgender and Gender Diverse Problems list (see Appendix B). We requested the members of the focus group by email to test the TGDP list prior to the start of this study. The research team discussed their rephrasing suggestions and incorporated those into the final list (Appendix B).

## Method

### Procedure and participants

We recruited TGD participants (age 18+) at Dutch centers for gender dysphoria between March 2022 and May 2023. Online TGD platforms, e.g. T-Forum and The Continuum, posted information on their sites to recruit participants. The promotional material provided information about the survey. A QR code enabled the participants to approach the questionnaires directly. We included the online questionnaires in the Open University**’**s secure research platform, O4U, to guarantee anonymity. Participants provided informed consent by ticking a box before starting the questionnaire. The TGDP list was part of a larger survey on the experiences of TGD people, the (self)stigmatization they perceived during transition, their coping behavior, and the perceived social support. The first questionnaire to complete was about demographic characteristics followed by the TGDP list. A total of 161 TGD individuals started the survey and 124 participants completed the TGDP list; the remaining 37 participants started the survey but did not answer the TGDP questions.

### General description

The researchers adapted the item wordings (see Appendix B, Transgender and Gender Diverse Problems List) to the question: “How often does this situation occur?” The TGDP list consists of cluster 1 Problems arising from the care process (CARE, 10 items), cluster 2 Consequences of the transition in relation to yourself (SELF, 14 items), cluster 3 Consequences of the transition in relation to others (OTHER, 7 items), and cluster 4 Problems arising from society (SOC, 16 items). The possible answers were: 1 = never, 2 = sometimes, and 3 = often. Cluster sum scores (CARE, SELF, OTHER, and SOC) were obtained by summing up item scores within each cluster.

The TGDP list is part of a longitudinal study of the Open University of the Netherlands about experiences of TGD people in transition.

### Statistical analyses

We provided descriptive statistics per age, gender identity, phase of transition, and education level using SPSS, version 29.0.0.0 (see Table 3). The relevant variables were checked for univariate outliers using boxplot inspection (+/− 3*SD*), and for multivariate outliers using Mahalanobis Distance (Tabachnik & Fidell, 2013). One univariate outlier was found with a very high score on SOC. We identified no multivariate outliers (*p* = .001). All cases were retained for analysis. We conducted several multivariate analyses of variance (MANOVA) to examine potential differences in the dependent variables between groups based on identity and phase of transition. We examined the influence of age on the experienced frequency scores of TGD problems by conducting multiple regression analyses between the cluster sum scores and age.

**Table 3.** Demographic characteristics and descriptive statistics of participants.

Demographic characteristics	*n*	*M (SD)*	%
Age	124	36.6 (15.0)	
Trans man	41	29.3 (11.8)	
Trans woman	55	43.1 (15.7)	
Nonbinary	17	33.3 (12.7)	
Other	11	36.3 (11.8)	
Gender identity			
Trans man	41		33.1
Trans woman	55		44.4
Nonbinary	17		13.7
Other	11		8.9
Phase of transition			
Pre	39		31.5
During	44		35.5
Post	41		33.1
Education level			
Primary education	3		2.4
Secondary education	22		17.7
Higher vocational education	39		31.5
Scientific education	50		40.3
Other	10		8.1

An a priori power analysis was conducted using G*Power version 3.1.9.7 (Faul et al., 2009) for required sample size estimation. The effect size was .35, considered to be large using Cohen**’**s (1988) criteria. With a significance criterion of *α* = .05 and power = .80, the minimum sample size needed with this effect size is *N* = 93 for MANOVA Global effects. Thus, the obtained sample size of *N* = 124 was adequate to answer the research questions.

## Results

### Demographic characteristics

Participants (*N*** **=** **124), aged 18–74 (*M*** **=** **36.58, *SD*** **=** **14.96), had the option to self-identify as trans man, man, trans woman, woman, or nonbinary, or other. In the latter case they could articulate their identity in their own words. The people who identified as others used descriptions such as *not woman*, *transmasculine*, or *nonbinary transmasculine.* They formed a small and heterogeneous group. We asked about gender assigned at birth and gave the answering options: male, female, or other. None of the participants registered as other. All participants identifying as trans woman were assigned male at birth. Most participants identifying as trans man were assigned female at birth; however, two trans men stated they were assigned male at birth. This identity ambiguity made us classify their identity as other (Table 3). The majority of the nonbinary participants (*n*** **=** **12) were assigned female at birth, the remainder (*n*** **=** **5) were assigned male at birth. The classification from Budge et al. (2013) was used to identify the phases of transition: pretransition, during transition, and posttransition. In the Dutch questionnaire we formulated the pretransition phase the *“*diagnostic*”* phase, when the TGD client meets with a psychologist or psychiatrist to talk about gender dysphoria and explore transition possibilities. The during transition phase was formulated the *“*hormonal*”* phase, when people undergo hormonal treatments or surgical operations, and the posttransition phase was formulated the *“*after transition*”* phase, when participants considered their transition completed (Table 3). Most participants were in transition at the time of the survey; 31.5% of the participants were in the pretransition phase and 35.5% were in the during transition phase. Participants were asked to report their highest achieved education level (primary education, secondary education, higher vocational education, scientific education or other, see Table 3).

### Frequency of experienced problems

We categorized the mean item scores per cluster and provided the overall mean scores per cluster, along with the mean scores per phase of transition and identity (see Appendix C, Table C1). We used a cut off score of *M_overall_* > 2.00 indicating that participants at least *“*sometimes*”* experienced the situation.

In cluster 1 Problems arising from the care process the highest overall mean scores were found on the items “The medical transition process takes a long time” (CARE1, *M*** **=** **2.50) and “Dependence on care providers for a decision in the transition process” (CARE3, *M*** **=** **2.33). Other high scoring items were: “Uncertainty about waiting times for surgery” (CARE2, *M*** ***=*** **2.27), “Diagnostics feels like an examination” (CARE7, *M*** **=** **2.19), and “Bureaucracy within the hospital” (CARE9, *M*** **=** **2.04).

In cluster 2 Consequences of the transition in relation to yourself the highest overall mean scores were found on the items “The transition takes a lot of energy” (SELF10, *M*** **=** **2.35) and “Shame for your own body that hinders intimate relationships” (SELF3, *M*** ***=*** **2.28). The items “Confronting your own body while showering” (SELF8, *M*** **=** **2.23) and “Finding it difficult to tell the family about the transition” (SELF4, *M*** **=** **2.15) also had high overall mean scores.

In cluster 3 Consequences of the transition in relation to others the highest overall mean scores were found on the items “Not daring to swim out of dissatisfaction with your body” (OTHER5, *M*** **=** **2.36), “Fear of dismissive reaction of others” (OTHER3, *M*** **=** **2.23), and “Finding it difficult to buy the right clothes” (OTHER7, *M*** ***=*** **2.02).

In cluster 4 Problems arising from society the highest overall mean scores were found on the items “Being addressed incorrectly on the phone, for instance as a trans woman with ‘sir’ and as a trans man with ‘madam’” (SOC8, *M*** **=** **2.31) and “People reacting dismissively to transgender people” (SOC9, *M*** **=** **2.05). The items “(Having to) explain the transition and being transgender over and over again” (SOC10, *M*** **=** **2.04), “Receiving unwanted questions about the transition of people you encounter” (SOC1, *M*** **=** **2.02), and “Choosing the “right” dressing room or toilet” (SOC13, *M*** **=** **2.00) also had high overall mean scores.

### Differences between gender identity subgroups

To address the second research question, we conducted a MANOVA to investigate potential differences among trans men, trans women, and nonbinary people on the items within the respective clusters. The identity category *other* was not used in the calculations because of the limited number of participants (*n*** **=** **11) and the internal gender variation within that group.

Table 4 displays only significant differences between trans men, trans women, and nonbinary people.

**Table 4.** ANOVAs related to identity.

		Test of between subjects effects			Post hoc				*M*		
Variable		*F*	*p*	*ŋ* ^2^	*p*	Lower bound	Upper bound	level	TM	TW	NB
Cluster 1	Problems arising from the care process										
	Pillai’s trace = .437, *F*(20,204) = 2.85, *p* <.001, *ŋ*^2^ = .22										
CARE8	Unfamiliarity with nonbinary in medical gender circuit	*F* (2,110) = 10.980	<.001	.17	<.001 <.001	−1.18 −1.33	−.36 −.54	TM vs. NB TW vs. NB	1.59	1.42	2.35
Cluster 2	Consequences of the transition in relation to yourself										
	Pillai’s trace = .390, F(28,196) = 1.70, p = .021, ŋ2 = .20										
SELF3	Shame for your own body that hinders intimate relationships	*F* (2,110) = 6.700	.002	.11	<.001 .026	.23 −.88	.84 −.06	TM vs. TW TW vs. NB	2.54	2.00	2.47
Cluster 3	Consequences of the transition in relation to others										
	Pillai’s trace = .397, F(14,210) = 3.71, p <.001, ŋ2 =.20										
OTHER2	Partner struggling with the transition	*F* (2,110) = 3.749	0.27	.064	.012	.11	.88	TW vs. NB	1.41	1.67	1.18
OTHER3	Fear of dismissive reaction of others	*F* (2,110) = 4.078	.020	.07	.005	.12	.69	TM vs. TW	2.46	2.05	2.18
OTHER5	Not daring to swim out of dissatisfaction with your body	*F* (2,110) = 8.568	<.001	.14	.003 <.001	.15 −1.13	.74 −.34	TM vs.TW TW vs. NB	2.54	2.09	2.82
OTHER6	Not knowing if you can say something about the transgender background on a date	*F* (2,110) = 5.129	.007	.09	.002	.19	.83	TM vs. TW	2.07	1.56	1.65
OTHER7	Finding it difficult to buy the right clothes	*F* (2,110) = 6.008	.003	.10	.004 .009	.15 −.99	.78 −.14	TM vs. TW TW vs. NB	2.20	1.73	2.29
Cluster 4	Problems arising from society										
	Pillai’s trace = .739, F(32,192) = 3.52, p < .001, ŋ2 = .37										
SOC1	Receiving unwanted questions about the transition of people you encounter	*F* (2,110) = 7.015	.001	.11	<.001	.26	.84	TM vs. TW	2.29	1.75	2.06
SOC4	Negative financial consequences of the transition	*F* (2,112) = 3.709	.028	.06	.024 .036	−.68 .03	−.05 .88	TM vs. TW TW vs. NB	1.56	1.93	1.47
SOC7	Being recognized as transgender when making new contacts	*F* (2,112) = 4.011	.021	.07	.008 .013	.15 .11	.98 .91	TM vs. NB TW vs. NB	1.80	1.75	1.24
SOC13	Choosing the appropriate dressing room or toilet	*F* (2,112) =8.899	<.001	.14	.003 <.001	−1.11 −1.33	−.23 −.48	TM vs. NB TW vs. NB	1.98	1.75	2.65

Note. TM: trans man; TW: trans woman; NB: nonbinary.

#### Cluster 1

On the item about “Unfamiliarity with nonbinary people in medical gender circuit” (CARE8) nonbinary people (*M*** **=** **2.35) scored higher than trans men (*M*** **=** **1.59) and trans women (*M*** **=** **1.42).

#### Cluster 2

On the item “Shame for your own body…” (SELF3), trans men (*M*** **=** **2.54) and nonbinary people (*M*** **=** **2.47) scored higher than trans women (*M*** **=** **2.00).

#### Cluster 3

On the item “Partner struggling with the transition” (OTHER2) trans women (*M*** **=** **1.67) scored higher than nonbinary people (*M*** **=** **1.18) and on the item “Fear of dismissive reaction …” (OTHER3) trans men (*M*** **=** **2.46) scored higher than trans women (*M*** **=** **2.05). On the item “Not daring to swim…” (OTHER5) trans men (*M*** **=** **2.54) and nonbinary people (*M*** **=** **2.82) scored higher than trans women (*M*** **=** **2.09). “Not knowing if you can say something about the transgender background on a date” (OTHER6) was scored higher by trans men (*M*** **=** **2.07) than by trans women (*M*** **=** **1.56). And on the item “Finding … the right clothes” (OTHER7) trans men (*M*** **=** **2.20) and nonbinary people (*M*** **=** **2.29) scored higher than trans women (*M*** **=** **1.73).

#### Cluster 4

The item “Receiving unwanted questions…” (SOC1) was scored higher by trans men (*M*** **=** **2.29) than by trans women (*M*** **=** **1.75). On the item “Negative financial consequences of the transition” (SOC4) trans women (*M*** **=** **1.93) scored higher than trans men (*M*** **=** **1.56) and nonbinary people (*M*** **=** **1.47). The item “Being recognized as transgender when making new contacts” was scored higher by trans men (*M*** **=** **1.80) and trans women (*M*** **=** **1.75) than by nonbinary people (*M*** **=** **1.24). And on the item about “Choosing the appropriate dressing room …” (SOC13) nonbinary people scored higher (*M*** **=** **2.65) than trans men (*M* = 1.98) and trans women (*M*** **=** **1.75).

### Differences between phases of transition

The third research question about experiencing differences with regard to the phases of transition only showed significant multivariate differences in cluster 1 (see Table 5; only significant differences are shown). On the item “Complications after surgery” (CARE6) TGD people scored higher post transition (*M*** **=** **1.61) than in the pretransition (*M*** **=** **1.21) and the during transition phase (*M*** **=** **1.32). On the item “Diagnostics feels like an examination” (CARE7) TGD people scored higher during transition (*M*** **=** **2.43) than post transition (*M*** **=** **1.98). On the item about “Bureaucracy within the hospital” (CARE9) TGD people in the during transition phase (*M*** **=** **2.25) and post transition (*M*** **=** **2.10) scored higher than TGD people in the pretransition phase (*M*** **=** **1.74).

**Table 5.** ANOVAs related to phase of transition.

Variable		Test of between subjects effects			Post hoc				*M*		
		*F*	*p*	*ŋ* ^2^	*p*	Lower bound	Upper bound	level	Pre	During	Post
Cluster 1	Problems arising from the care process										
	Pillai’s trace = .280, F(20,226 = 1.84), p = .018, partial ŋ2 =.14										
CARE6	Complications after surgery	*F*(2,121) = 5.119	.007	.08	.003 .024	−.66 −.54	−.14 −.04	D vs. A H vs. A	1.21	1.32	1.61
CARE7	Diagnostics feels like an examination	*F*(2,121) = 3.140	.047	.05	.014	.09	.82	H vs. A	2.15	2.43	1.98
CARE9	Bureaucracy within the hospital	*F*(2,121) = 4.670	.011	.07	.003 .041	−.84 −.69	−.17 −.01	D vs. H D vs. A	1.74	2.25	2.10

*Note.* Pre: pretransition; During: during transition; Post: posttransition.

### Associations with age

Multiple regression analysis was used to answer the fourth research question concerning the association between age and the experienced frequency of problems. Age was used as a criterion variable, and CARE (sum scores cluster 1), SELF (sum scores cluster 2), OTHER (sum scores cluster 3), and SOC (sum scores cluster 4) were used as predictor variables. The regression model was statistically significant (*R*^2^ =.085, F (4, 119) = 3.850, *p* =.006), indicating that problems happened more often with younger age. The results of SELF (*β* = −0.609, *p* = .046) significantly contributed to the model. CARE (*β* = −0.585, *p* = .091), OTHER (*β* = −0.144, *p* = .832), and SOC (*β* = −0.102, *p* = .729) did not contribute.

## Discussion

This study, to our knowledge, provided the first inventory of the problems experienced by TGD people during the various phases of transition. Our goal was to determine the frequency of experiencing these problems and to examine whether gender identity, transition stage and age were related to these experiences. We found that age was influential; younger TGD people experienced the problems more frequently, especially those related to cluster 2 Consequences of transition in relation to yourself. Gender identity also played a role in some of the problems, but the stage of transition did not play a significant role in most problems. Our study provides a complement to existing research mentioned above, because we examined everyday problems that TGD people may experience during the various stages of transition.

The study revealed that the most frequently encountered problem situations during transition were the long waiting lists, the dependence on care providers, the confrontation with one**’**s body while showering or swimming, the energy it takes to go in transition, misgendering, and the fear of dismissive reactions and rejection. The experiences with long waiting lists were in line with findings of De Bekker et al. (2019) and Quak (2010) and research of the Dutch patient organization Transvisie (Van den Boom, 2016). The waiting lists increased since the outburst of the COVID pandemic in 2020 which led to fewer visitors at the gender clinics. Dependency on care providers aligns with the findings of Vogelsang et al. (2016) who found that TGD people felt dependent on the healthcare staff. Misgendering, a high scoring item in our survey, was in previous research also found to take place in the public environment, at the workplace, and in gender-inclusive healthcare settings (Chang et al., 2023; McNamarah, 2021). Misgendering may lead to decreased body-identity congruence and increased body dissatisfaction (Mitchell et al., 2021). The lack of adequate information, the fear of rejection, and worries about treatments, worded in some TGDP items, were also found by Puckett et al. (2018) in their research into barriers to gender affirming care for transgender people. In addition, our results revealed other frequently experienced TGD problems, such as the large amount of energy it takes to go into transition, the difficulty to tell the family about the transition, and that diagnostics feels like an examination.

We found differences between the experiences of trans men, trans women, and nonbinary people related to the perceived frequency of the TGD problems. Some differences were obvious; compared to trans men and trans women, nonbinary people were most often confronted with unfamiliarity with nonbinary in the medical gender circuit. Other differences, such as those related to body satisfaction, were less evident. Noticeable were the higher scores of trans men and nonbinary people versus trans women on the items “Not daring to swim…” and “Shame of your own body.” Trans men and nonbinary people scored high on the clothing fit issues demonstrating the difficulties TGD people may experience when their changing physique has not (yet) an adequate fit with standard cisgender sizes in fashion (Reilly et al., 2019). The disparities between the experiences of trans men, trans women and nonbinary people were visible with regard to reactions from the environment, the experience of one**’**s own body, and financial consequences of the transition. Our findings underline those of Tatum et al. (2020). They found that nonbinary people**’**s concern with living full time in their desired gender was different from the concern of trans men and trans women: nonbinary people lived full time in their desired gender prior to coming out. As a result, nonbinary people may experience fewer problems with stigmatization. However, nonbinary people experienced lower social support from family and friends than trans men and trans women did (Aparicio-García et al., 2018). The variations between groups of participants in our study also underline the observations by De Bekker et al. (2019), who revealed a heterogeneous group of TGD people in their report about the “bottlenecks in transgender care.”

The regression model showed that problems were related to these experiences more often at younger ages. Young TGD adults are at an increased risk for self-harm, suicidality, and weight-related problems (Peterson et al., 2017). These results show how important it is to help younger TGD people in transition (De Vries & Cohen-Kettenis, 2012). Tatum et al. (2020) also reported differences in age regarding the experiences of trans men, trans women, and nonbinary people; transgender people reported thinking about their gender identity earlier in life than nonbinary people, which may mean they experience problems earlier than nonbinary people.

The outcomes of this study can be used by healthcare professionals in diagnostic and counseling interviews before and during the transition process to discuss the client**’**s expectations and potential challenges they may encounter. This can lead to more realistic expectations and consequently improve the treatment outcomes (Laferton et al., 2017). Future research is recommended to understand the role of expectation management regarding the course of the transition process taking into account aspects such as age and gender identity. The TGDP list can also serve as a basis for developing a diagnostic coping questionnaire regarding the problems they experience during transition. The TGDP list can be a tool for policymakers to create policies that are more focused on people with TGD backgrounds because (re)knowing the problems can provide an opportunity to improve a situation.

## Strengths and limitations

The moderate sample size presents a limitation. This may have been the result of the decreased visit to the gender clinics during the COVID crisis reducing the possibility of bringing promotional materials to the attention of potential participants. The length of the complete questionnaire, necessary for follow-up research, may have caused fewer people to complete the list. The statements that were not consistently worded using inclusive language, may also have been a reason for not completing the questionnaire. When administering the questionnaires, we asked participants whether they were in the diagnostic phase, the hormonal phase or the after transition phase. In doing so, we did not clearly formulate what was meant by these phases. This may have led to misunderstandings when completing the questionnaire.

Because of the rapidly changing world of TGD people, we recommend monitoring these changes. The results of this study cannot be generalized to other sociocultural contexts; the problems experienced by Dutch TGD people may be different from those in other (non-western) countries.

A strength of this study is the involvement of TGD people as co-creators in the developmental process of the TGDP list. Another strength of this study is the mapping of the TGD specific problem situations with which we sketched a full picture of the problems TGD people may experience during transition. The TGDP list can serve as discussion topic during counseling. The next step might be to study how TGD people cope with these specific problems. Therefore, we recommend follow-up research on this topic.

## Ethical approval

All procedures performed in the study involving human participants were in accordance with the ethical standards of the ethical committee of the Open University of The Netherlands and with the 1964 Helsinki Declaration and its later amendments or comparable ethical standards.

## Appendix A.

**Table:** Statements per cluster.

		*M*	*SD*
**Cluster 1. Problems arising from the care process** (*M =* 3.52)			
10.	The medical transition process takes a long time	4.54	0.78
70.	Waiting times are long and criteria are unclear	4.31	1.32
26.	Uncertainty about waiting times for surgery	4.00	0.82
21.	Dependence of care providers	3.85	1.21
57.	Lack of psychological aftercare	3.85	1.07
35.	Slow progress in treatment	3.77	0.83
67.	Doctors do not listen properly to your wishes	3.77	1.42
40.	Complications after surgery	3.67	1.15
22.	Diagnostics feels like an examination	3.54	0.97
72.	Not enough experts with experience in providing assistance	3.15	1.34
32.	Uncertainty about medical aftercare by the gender team after transition	3.08	1.44
55.	Unfamiliarity with non-binarity in medical gender circuit	2.92	1.80
17.	Bureaucracy within the hospital	2.85	1.28
31.	Problems with reimbursement by health insurance companies	2.77	1.30
54.	Insurance does not pay for testosterone injections	2.69	1.55
**Cluster 2. Consequences of the transition in relation to yourself** (*M* = 3.28)			
11.	Personal doubt, fear and confusion	4.69	0.63
3.	Fear of losing friends and family	4.15	0.90
28.	Shame of your own body hinders embarking on intimate relationships	4.15	1.07
13.	How do you tell your family about your transition	3.92	1.26
53.	Fear of the consequences of the operation	3.77	1.17
36.	Uncertainty about the mental effects of medical treatment	3.46	1.20
37.	Uncertainty about the effects of the transition on physical appearance	3.31	1.25
48.	Loss of sexual sensitivity after genital surgery	3.31	1.44
4.	Confronting your body while showering	3.23	1.01
12.	Fear of making the wrong choice about surgery	3.08	1.55
69.	The transition takes a lot of energy	3.08	1.31
23.	Disappointment over the missed past in the desired gender identity	3.08	1.61
19.	Choice of the right moment to live in the desired gender identity	3.00	1.47
25.	Changes in sexual desire due to hormone treatment	2.92	1.12
52.	Choice of whether or not to opt for surgery	2.92	1.38
64.	Reduced fertility due to treatment	1.92	1.04
47.	Loss of head hair	1.77	1.24
**Cluster 3. Consequences of the transition in relation to others** (*M* = 2.99)			
27.	Losing friends	3.92	1.12
59.	Partner not accepting the transition	3.85	1.07
30.	Fear of rejection	3.50	1.17
75.	Partner cannot keep up with the rapid progress of the transition	3.46	0.97
49.	Loss of sexuality within relationships	3.15	1.34
65.	Not being brave enough daring to swim because of appearance	3.00	1.08
42.	What do I wear and what suits me best?	2.85	1.34
63.	Not to know whether to tell about your “trans” background while on a date	2.69	1.18
1.	At the beginning of transition it is difficult to buy clothes	2.38	1.12
68.	Finding suitable clothes	2.38	1.33
14.	Not looking age-appropriate	1.77	1.36
6.	Impertinent questions about the transition by people you encounter	3.77	1.24
45.	Job loss	3.62	1.50
58.	Be laughed at in the street	3.54	1.39
44.	Others finding it difficult to change the gender used in speech	3.38	1.19
56.	Deliberately being misgendered	3.31	1.38
**Cluster 4. Problems arising from society** (*M =* 2.67)			
73.	Being discriminated against	3.31	1.49
33.	Annoying to be recognized as transgender when making new contacts	3.08	1.19
34.	The expectation of stereotype gender norms when you are in transition	3.08	0.99
66.	The world thinking in terms of “man” or “woman”	3.00	1.47
5.	Being misgendered on the phone	2.92	0.95
7.	People having specific ideas about harmfulness of transgender people (keeping transgender people away from customers or children)	2.92	1.19
16.	Being groped in the crotch	2.85	1.72
43.	Society suddenly giving you a different status as woman	2.85	1.34
8.	People being afraid that transgender people will abuse children	2.77	1.24
60.	People thinking that gender dysphoria is just a phase in life	2.77	1.54
41.	Constantly explaining who you are	2.69	1.49
51.	Being expected to explain your sexual orientation	2.54	1.33
74.	Things are plain and simple for you, but not for the outside world	2.54	1.39
18.	Difficulty in registering your house in your own name after transition	2.46	1.33
50.	People connecting sexuality with gender	2.46	1.20
62.	Being told: “You turned out well”	2.46	1.56
29.	It is difficult to change dressing rooms at the gym	2.38	1.45
61.	The public transport worker not accepting your public transport card.	2.38	1.12
2.	Choosing a toilet is difficult at the start of transition	2.31	1.25
20.	Being asked: “When did you decide to be transgender?’	2.15	1.21
39.	Traveling abroad is difficult because of low tolerance for transgender people.	2.15	1.14
24.	People want to discuss gender neutrality with you	2.08	1.12
9.	People are afraid transgender people will contaminate children	2.00	1.29
38.	Being searched by Customs	1.69	0.75
15.	The hairdresser refusing to cut your hair how you want	1.62	0.65
71.	People making lengthy apologies when they speak	1.62	0.96
**Cluster 5. Financial consequences of the transition**. (*M* = 3.46)			
46.	Financial consequences of the transition	3.46	1.13

*Note.* Focus question: “Which difficult situations are transgender people confronted with during the transition process? .”

## Appendix B.

**Table:** Transgender and gender Diverse problems list.

Code	Variables
*Cluster 1*	*Problems arising from the care process*
CARE1	The medical transition process takes a long time
CARE2	Uncertainty about waiting times for surgery
CARE3	Dependence on care providers for a decision in the transition process
CARE4	Lack of psychological aftercare
CARE5	My wishes are not being listened to properly
CARE6	Complications after surgery
CARE7	Diagnostics feels like an examination
CARE8	Unfamiliarity with nonbinary people in medical gender circuit
CARE9	Bureaucracy within the hospital
CARE10	Not being (fully) reimbursed for treatment by health insurers
*Cluster 2*	*Consequences of the transition in relation to yourself*
SELF1	Doubt, fear and confusion about the transition
SELF2	Fearing the loss of friends and family due to the transition
SELF3	Shame for your own body that hinders intimate relationships
SELF4	Finding it difficult to tell the family about the transition
SELF5	Fearing the consequences of a possible operation
SELF6	Uncertainty about the psychological effects of medical treatment
SELF7	Uncertainty about the effects of the transition on appearance
SELF8	Confronting your own body while showering
SELF9	Fear of making the wrong choice regarding the operation
SELF10	The transition takes a lot of energy
SELF11	Choosing the right time to start living in the opposite sex
SELF12	Changes in sexual desire due to the use hormones
SELF13	Making the choice between surgery or not
SELF14	Thoughts on reduced fertility due to the treatment
*Cluster 3*	*Consequences of the transition in relation to others*
OTHER1	Losing friends because of the transition
OTHER2	Partner struggling with the transition
OTHER3	Fear of dismissive reaction of others
OTHER4	Fear of loss of sexuality within the relationship
OTHER5	Not daring to swim out of dissatisfaction with your body
OTHER6	Not knowing if you can say something about the transgender background on a date
OTHER7	Finding it difficult to buy the right clothes
*Cluster 4*	*Problems arising from society*
SOC1	Receiving unwanted questions about the transition of people you encounter
SOC2	Job loss due to the transition
SOC3	Being laughed at on the street
SOC4	Negative financial consequences of the transition
SOC5	Others deliberately continuing to use your former name
SOC6	Discrimination based on being transgender
SOC7	Being recognized as transgender when making new contacts
SOC8	Being addressed incorrectly on the phone, for instance as a trans woman with "sir" and as a trans man with "madam"
SOC9	People reacting dismissively to transgender people
SOC10	(Having to) explain the transition and being transgender over and over again
SOC11	Getting questions about sexual orientation in combination with being transgender
SOC12	Being told: “You turned out well”
SOC13	Choosing the appropriate dressing room or toilet
SOC14	Problems on public transport while traveling with a public transport card
SOC15	Being asked: “When did you decide to be transgender?”
SOC16	Traveling abroad is difficult due to lower tolerance abroad

## Appendix C

**Table C1.** Mean frequency of experience of problems by phase of transition and identity per cluster.

Code	Cluster	MEAN							
		Overall (*n* = 124)	Pre (*n* = 39)	During (*n* = 44)	Post (*n* = 41)	TM *(n* = 41)	TW (*n* = 55)	NB (*n* = 17)	OTHER (*n* = 11)
*1. Problems arising from the care process*									
CARE1	The medical transition process takes a long time	2.50*	2.56	2.52	2.41	2.41	2.55	2.53	2.55
CARE2	Uncertainty about waiting times for surgery	2.27*	2.18	2.43	2.20	2.29	2.22	2.29	2.45
CARE3	Dependence on care providers for a decision in the transition process	2.33*	2.26	2.50	2.22	2.41	2.16	2.53	2.55
CARE4	Lack of psychological aftercare	1.53	1.33	1.59	1.66	1.66	1.44	1.41	1.73
CARE5	My wishes are not being listened to properly	1.63	1.46	1.61	1.80	1.68	1.56	1.47	2.00
CARE6	Complications after surgery	1.38	1.21	1.32	1.61	1.46	1.33	1.29	1.45
CARE7	Diagnostics feels like an examination	2.19*	2.15	2.43	1.98	2.32	2.04	2.35	2.27
CARE8	Unfamiliarity with non-binary in medical gender circuit	1.67	1.56	1.73	1.71	1.59	1.42	2.35	2.18
CARE9	Bureaucracy within the hospital	2.04*	1.74	2.25	2.10	2.15	1.89	2.18	2.18
CARE10	Not being (fully) reimbursed for treatment by health insurers	1.81	1.72	1.93	1.78	1.66	2.04	1.65	1.55
*2. Consequences of the transition in relation to yourself*									
SELF1	Doubt. fear and confusion about the transition	1.76	1.92	1.64	1.73	1.73	1.80	1.82	1.55
SELF2	Fearing the loss of friends and family due to the transition	1.98	1.97	2.00	1.98	1.95	2.07	1.82	1.91
SELF3	Shame for your own body that hinders intimate relationships	2.28*	2.44	2.30	2.12	2.54	2.00	2.47	2.45
SELF4	Finding it difficult to tell the family about the transition	2.15*	2.31	2.11	2.05	2.37	2.11	2.00	1.82
SELF5	Fearing the consequences of a possible operation	1.77	1.62	1.82	1.85	1.80	1.73	1.65	2.00
SELF6	Uncertainty about the psychological effects of medical treatment	1.52	1.62	1.52	1.41	1.61	1.38	1.71	1.55
SELF7	Uncertainty about the effects of the transition on appearance	1.90	2.03	1.89	1.78	1.78	1.95	1.82	2.18
SELF8	Confronting your own body while showering	2.23*	2.23	2.34	2.12	2.41	2.11	2.29	2.09
SELF9	Fear of making the wrong choice regarding the operation	1.56	1.44	1.59	1.63	1.61	1.45	1.53	1.91
SELF10	The transition takes a lot of energy	2.35*	2.15	2.45	2.44	2.49	2.20	2.41	2.55
SELF11	Choosing the right time to start living in the opposite sex	1.77	1.97	1.66	1.68	1.71	1.82	1.76	1.73
SELF12	Changes in sexual desire due to the use of hormones	1.50	1.26	1.64	1.59	1.54	1.44	1.53	1.64
SELF13	Making the choice between surgery or not	1.61	1.67	1.66	1.51	1.66	1.49	1.76	1.82
SELF 14	Thoughts on reduced fertility due to the treatment	1.28	1.31	1.25	1.29	1.29	1.31	1.12	1.36
		Overall (*n* = 124)	Pre (*n* = 39)	During (*n* = 44)	Post (*n* = 41)	TM (*n* = 41)	TW (*n* = 55)	NB (*n* = 17)	Other (*n* = 11)
*3. Consequences of the transition in relation to others*									
OTHER1	Losing friends because of the transition	1.62	1.56	1.59	1.71	1.56	1.80	1.35	1.36
OTHER2	Partner struggling with the transition	1.49	1.59	1.41	1.49	1.41	1.67	1.18	1.36
OTHER3	Fear of dismissive reaction of others	2.23*	2.23	2.25	2.20	2.46	2.05	2.18	2.27
OTHER4	Fear of loss of sexuality within the relationship	1.43	1.51	1.39	1.39	1.46	1.47	1.29	1.27
OTHER5	Not daring to swim out of dissatisfaction with your body	2.36*	2.33	2.50	2.24	2.54	2.09	2.82	2.36
OTHER6	Not knowing if you can say something about the transgender background on a date	1.77	1.51	1.82	1.98	2.07	1.56	1.65	1.91
OTHER7	Finding it difficult to buy the right clothes	2.02*	1.92	2.09	2.02	2.20	1.73	2.29	2.36
*4. Problems arising from society*									
SOC1	Receiving unwanted questions about the transition of people you encounter	2.02*	1.74	2.16	2.12	2.29	1.75	2.06	2.27
SOC2	Job loss due to the transition	1.40	1.31	1.36	1.51	1.29	1.55	1.18	1.36
SOC3	Being laughed at on the street	1.54	1.54	1.57	1.51	1.49	1.62	1.41	1.55
SOC4	Negative financial consequences of the transition	1.72	1.77	1.68	1.71	1.56	1.93	1.47	1.64
SOC5	Others deliberately continuing to use your former name	1.61	1.56	1.68	1.59	1.66	1.58	1.59	1.64
SOC6	Discrimination based on being transgender	1.81	1.59	1.93	1.88	1.78	1.80	2.00	1.64
SOC7	Being recognized as transgender when making new contacts	1.70	1.51	1.89	1.68	1.80	1.75	1.24	1.82
SOC8	Being addressed incorrectly on the phone. for instance as a trans woman with "sir" and as a trans man with "madam"	2.31*	2.23	2.48	2.20	2.10	2.35	2.53	2.55
SOC9	People reacting dismissively to transgender people	2.05*	1.85	2.14	2.15	2.10	1.93	2.29	2.09
SOC10	(Having to) explain the transition and being transgender over and over again	2.04*	1.87	2.23	2.00	2.05	1.91	2.24	2.36
SOC11	Getting questions about sexual orientation in combination with being transgender	1.97	1.90	2.11	1.88	2.02	1.85	2.18	2.00
SOC12	Being told: “You turned out well”	1.65	1.36	1.61	1.95	1.68	1.69	1.41	1.64
SOC13	Choosing the appropriate dressing room or toilet	2.00*	2.13	2.09	1.78	1.98	1.75	2.65	2.36
SOC14	Problems on public transport while traveling on public transport card	1.26	1.15	1.27	1.34	1.39	1.15	1.41	1.09
SOC15	Being asked: “When did you decide to be transgender?”	1.61	1.41	1.73	1.68	1.68	1.53	1.82	1.45
SOC16	Traveling abroad is difficult due to lower tolerance abroad	1.87	1.72	2.02	1.85	1.83	1.78	2.18	2.00

*Note.* Pre: pretransition; During: during the transition; Post: posttransition; TM: trans man; TW: trans woman; NB: nonbinary; Other: other identity e.g. genderqueer.

*Highest scoring items per cluster.
